# Expanding the Genetic Code of *Xenopus laevis* Embryos

**DOI:** 10.1021/acschembio.3c00686

**Published:** 2024-01-26

**Authors:** Wes Brown, Lance A. Davidson, Alexander Deiters

**Affiliations:** † Department of Chemistry, University of Pittsburgh, Pittsburgh, Pennsylvania 15260, United States; ‡ Departments of Bioengineering, Developmental Biology, and Computational and Systems Biology, University of Pittsburgh, Pittsburgh, Pennsylvania 15260, United States

## Abstract

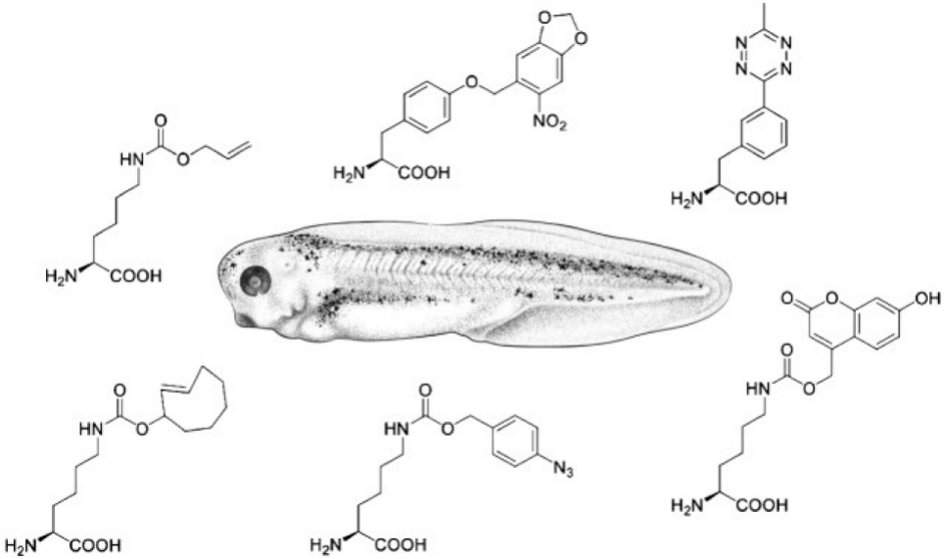

The incorporation of unnatural amino acids into proteins through genetic code expansion has been successfully adapted to African claw-toed frog embryos. Six unique unnatural amino acids are incorporated site-specifically into proteins and demonstrate robust and reliable protein expression. Of these amino acids, several are caged analogues that can be used to establish conditional control over enzymatic activity. Using light or small molecule triggers, we exhibit activation and tunability of protein functions in live embryos. This approach was then applied to optical control over the activity of a RASopathy mutant of NRAS, taking advantage of generating explant cultures from *Xenopus*. Taken together, genetic code expansion is a robust approach in the *Xenopus* model to incorporate novel chemical functionalities into proteins of interest to study their function and role in a complex biological setting.

## Introduction

Genetic code expansion (GCE) is the precise installation of unique and functional amino acids into proteins that go beyond the canonical 20 side chains commonly seen in nature.^1−4^ The chemistries of these unnatural amino acids (UAAs) enable new biological function, from conditional control of enzyme function to bioorthogonal labeling.^5−7^ This is made possible by an orthogonal pyrrolysyl tRNA synthetase (PylRS) and tRNA (PylT) pair from the archaea *M. barkeri* or *M. mazei*.^4,8−12^ A PylRS that recognizes a UAA is generated through selections from a library of synthetases with mutated amino acid binding sites.^11−14^ The PylT anticodon naturally recognizes the amber stop codon (TAG) and is routinely used for GCE since it does not compete with any acylated tRNA and is also the least frequent stop codon in both pro- and eukaryotic cells.^15,16^ The PylRS/PylT system has been shown to not cross react with bacterial or eukaryotic synthetases or tRNA, allowing for reliable and robust installation of the UAA at an introduced amber stop codon in the protein of interest.^11−13^ Undesired amber suppression at stop codons in endogenous genes was undetectable in mammalian cells and *E. coli*.^17,18^ Release factors outcompete PylT by 3 orders of magnitude at endogenous amber stop codons, likely due to the surrounding sequence context having been evolved to prevent mistranslation.

We and others have successfully completed genetic code expansion experiments in different cell types and organisms with no or minimal effect on cell/animal health. First established in *E. coli*, the approach has since been expanded to mammalian cells,^19^*C. elegans*,^20^ flies,^21^ mice,^22^ and zebrafish.^9,23^ Many chemical functionalities were incorporated into proteins. Caged analogues of lysine or tyrosine have replaced protein active site residues in order to establish conditional control over their activity;^2^ photo-cross-linkers have been utilized to probe protein–protein interactions;^24^ and biorthogonal ligation handles have been placed onto proteins for labeling with fluorescent probes.^18^

We had recently adapted GCE to zebrafish embryos by injecting mRNA for the PylRS and protein of interest with an amber stop codon, along with the PylT and UAA directly into the fertilized oocyte.^25−27^ Injection of acylated tRNA into unfertilized *Xenopus laevis* oocytes for site-specific incorporation of a UAA has been utilized before,^28−30^ but protein production is limited and the synthesis of the acylated tRNA is challenging. Thus, to achieve robust UAA-incorporated protein expression in the developing frog embryo, we conducted GCE through the addition of a tRNA/tRNA synthetase pair to the protein biosynthetic machinery. Much of what we know about vertebrate early development comes from studies of the *Xenopus* model.^31^ Distinguishing factors of the frog embryo that make it a useful animal model are its amenability to explant culture for studying early developmental biology, predictable fate mapping of cells at early blastula stages, and their unique three chambered heart, which, unlike the zebrafish heart, contains an atrial septum, allowing for the study of septal defects during development.^32,33^ Their large size and high protein production also makes them amenable to proteomic studies.^34^ Thus, we translated GCE to *Xenopus* embryos for the first time.

## Results and Discussion

### Optimizing Genetic Code Expansion for the Developing *Xenopus* Embryo

**Figure 1:**
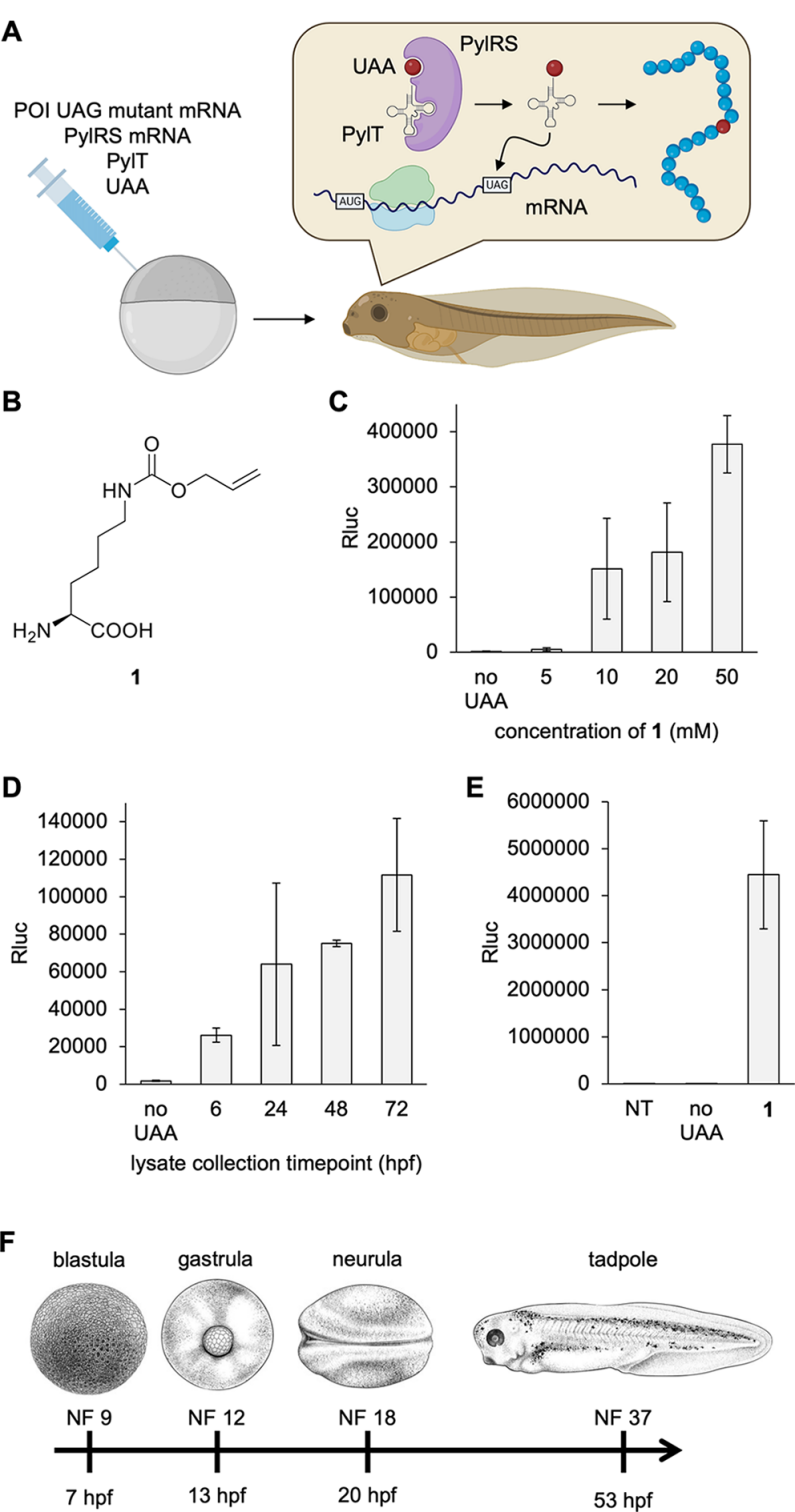
Genetic code expansion in *Xenopus*. (A) Injection and UAA incorporation into a protein of interest (POI) in embryos. (B) Structure of **1**. (C) Titration of **1** in the injection solution and incorporation into Rluc95UAG. (D) Incorporation of **1** (10 mM) over the first 72 h of development. (E) Incorporation of **1** into Rluc95UAG at 16 °C. All bars are means, and error bars are standard deviations from three embryos. (F) Nieuwkoop Faber (NF) stages of *Xenopus* embryos at certain time points at 23 °C. Panel F reproduced with permission from ref (37). Copyright 2022 The Company of Biologists. NT = nontreated embryo control. hpf = hours post-fertilization.

Incorporation of UAAs into proteins in *Xenopus* embryos built onto related experiments in zebrafish embryos.^9,25,27^ First, mRNA was generated for PylRS and the protein of interest with an amber stop codon at the desired site of UAA incorporation. This is accomplished by cloning the gene of interest into a pCS2 vector (Supporting Figure S1), a commonly used plasmid that acts as a template for Sp6 *in vitro* transcription and includes a 3′ SV40 polyA signal. The mMessage mMachine *in vitro* Transcription Kit (Thermo) was used to generate 5′ capped mRNA. The PylT was transcribed by using a PCR product as the template for T7 *in vitro* transcription. To test the GCE in *Xenopus* embryos, we utilized a *Renilla* luciferase reporter with a permissive surface leucine mutated to an amber stop codon (Rluc L95TAG). Luciferase activity is only present when the amber stop codon is suppressed, allowing for translation of full-length protein.^25^ A mixture containing PylRS mRNA, PylT, Rluc95UAG mRNA, and UAA was injected into fertilized one-cell-stage *Xenopus* embryos (Figure 1A). An alloc-protected lysine **1** (Figure 1B) was used to optimize conditions for incorporation into the Rluc reporter. Zebrafish studies showed efficient UAA incorporation and protein production with injection of equivalent amounts of PylRS mRNA and the gene of interest mRNA, along with PylT at the highest concentration that does not show toxicity (∼3–7 μg/μL). *Xenopus* protocols recommend starting with injecting 1 ng total of mRNA for assessing protein expression.^35^ We found that injecting this much was embryonically lethal, but that injecting 250 pg of PylRS mRNA and 250 pg of the Rluc 95UAG mRNA, along with an excess of 7.5 ng of PylT (combined in 5 nl total injection volume) into the cell of a fertilized embryo at the one-cell stage was well tolerated, resulting in normally developing embryos. For incorporation of **1**, we saw excellent expression of Rluc when it was included at a concentration of at least 10 mM in the injection solution (at least 50 pmol total injected, Figure 1C). While higher concentrations (20–50 mM) of **1** showed better incorporation efficiency, most UAAs are not soluble at those high concentrations in water. However, we have routinely made injection solutions of several UAAs at 10 mM in water for zebrafish injection without precipitation or needle clogging. In *Xenopus* embryos, the final concentration of UAA after injection would be roughly 50 μM (based on an injection volume of 5 nL and a cell volume of ∼1 μL^36^). Expression levels of the Rluc reporter were high by 24 hours post-fertilization (hpf) at RT and persisted at 72 hpf (Figure 1D). Many important developmental processes occur within these first 72 h, as gastrulation and establishment of cell fates is complete around 12 hpf, development of the spinal cord (neurulation) occurs by 22 hpf, and development of organs such as the heart have begun and continue to mature by 72 hpf.^37^ Rluc has a short half-life of about 4.5 h,^38^ meaning that incorporation of the UAA is still actively occurring over those 72 h. *Xenopus* develop into larvae with functioning organs within these 72 h; thus, expression of UAA-incorporated protein can be used to study any stage of early embryo development. A benefit of *Xenopus* embryos is that they can be incubated at lower temperatures to slow down development for experimental convenience without ill effects on embryo health.^35^ Incubating injected embryos at 16 °C showed excellent incorporation of **1** at 24 hpf (Figure 1E). This is useful for timing experiments to particular developmental stages. In our case, 24 hpf at 23 °C corresponds to stages 21−22 (end of neurulation), while 24 hpf at 16 °C corresponds to stages 11−12 (end of gastrulation, Figure 1F).

### Incorporation of Structurally and Functionally Diverse UAAs into Protein in the *Xenopus* Embryo

**Figure 2:**
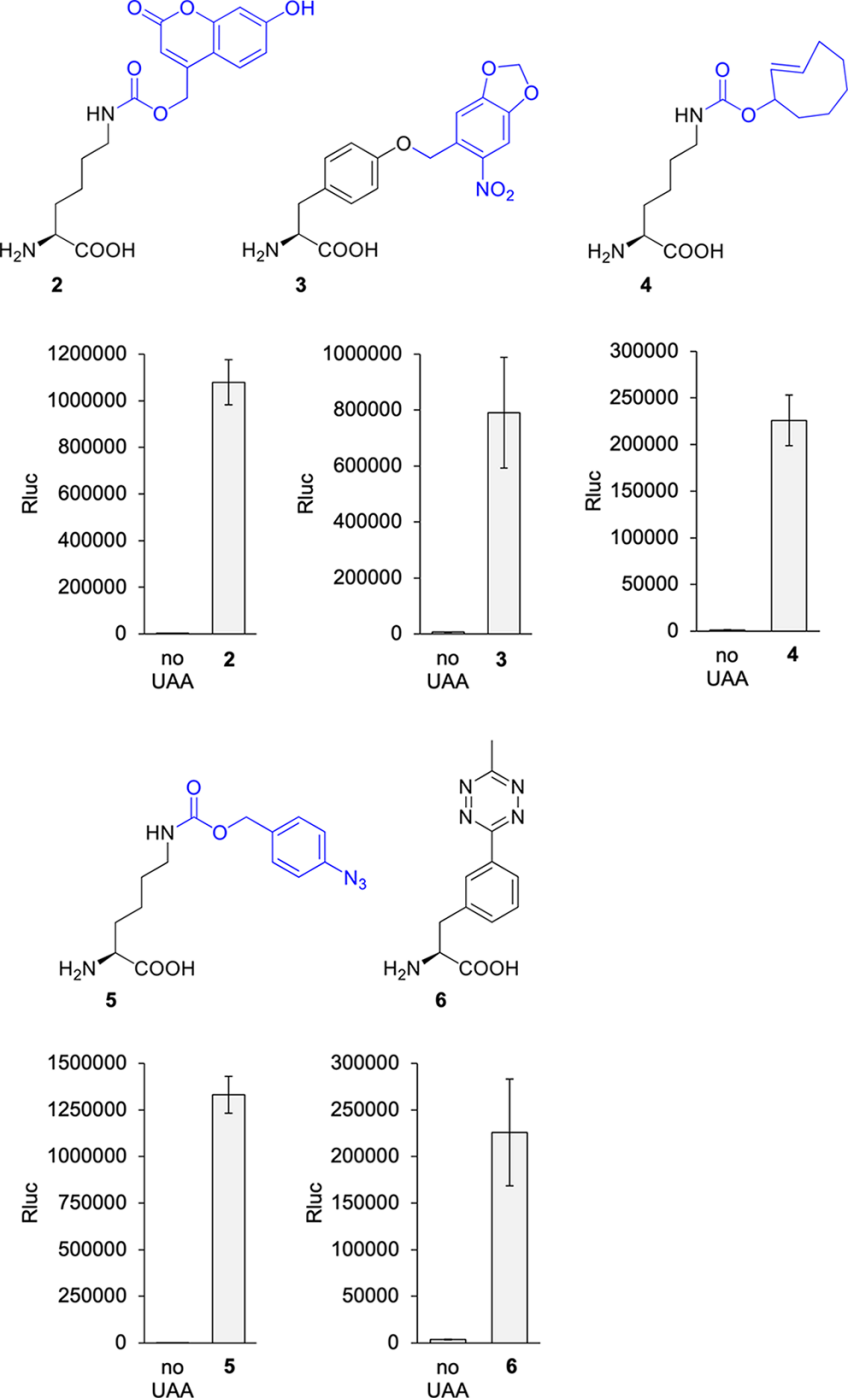
Genetic encoding of UAAs **2**–**6** in *Xenopus* embryos. Structures of **2**–**6**. Blue represents caging groups that are removed after treatment with 405 nm light or a small molecule trigger. Incorporation of **2**–**6** into the luciferase reporter was accomplished after injection of all components, including **2**, **4**, and **5**, while UAAs **3** and **6** were added to the media. Embryos were incubated at 23 °C, and assays were conducted at 24 hpf. Bars represent means, and error bars represent standard deviations of measurements from three independent embryos.

In order to demonstrate the broad utility of GCE in this animal model, we attempted incorporation of several UAAs with unique functions into the luciferase reporter using universal conditions: 250 pg of PylRS mRNA, 250 pg of luciferase reporter mRNA, 7.5 ng of PylT, 50 pmol of UAA per embryo, and 23 °C incubation until 24 hpf (Figure 2). UAAs **2** and **3** are photocaged variants of lysine and tyrosine, respectively, both of which have been used to optically control a wide variety of enzymes, including polymerases, recombinases, nucleases, kinases, phosphatases, and others.^39−41^ Both have demonstrated rapid decaging following brief exposure to UV or blue light, permitting time-resolved studies of signaling pathways or perturbation of zebrafish embryo development.^6,23^ The spatial resolution provided by optical control also allows for activation of protein function in specific cell populations of a whole organism.^42^ UAAs **4** and **5** are small molecule activated analogs of lysine. The *trans*-cyclooctene and *para*-azidobenzyl-containing caging group are rapidly cleaved through treatment with tetrazines or phosphines, respectively.^43,44^ These too have been used for conditional control of enzyme function and offer an orthogonal method for protein activation versus that of light-control.^43,45−47^ This has allowed for conditional activation of proteins in animal models such as the mouse where light penetration is limited.^47^ Because of the caging capability of these UAAs, mRNA of dual luciferase reporters (FlucK529UAG-Rluc for lysine analogues and Fluc340UAG-Rluc for the tyrosine analogue) were synthesized to assess both incorporation (resulting in Rluc expression) and decaging (resulting in activation of Fluc activity, as discussed in detail below). UAA **6** is a tetrazine-containing analogue of phenylalanine, which can be used in rapid bioconjugation reactions for post-translational labeling or pull-down of proteins.^48−50^ It was incorporated into the same Rluc95UAG reporter as **1**.

Injection of **2**, **4**, or **5**, together with their matching PylT/tRNA pair, led to effective incorporation into the luciferase reporter. While **1** expectedly had the highest incorporation efficiency with luminescence readings up to 1714-fold higher than background readings in the absence of the UAA, **2**, **4**, and **5** also had exceptional incorporation with 339-, 189-, and 1183-fold increases in luminescence above the background, respectively. To put this in perspective, in zebrafish embryos, we saw incorporation of **1** and **2** into the Rluc 95UAG reporter with a 217- and 70-fold increase in luminescence, respectively.^23^ Therefore, *Xenopus* is very efficient at incorporating these UAAs into luciferase reporters. This highly efficient reprogramming of UAG codons for unnatural amino acid incorporation may be attributed to the excellent orthogonality of the PylRS system in *Xenopus* (as demonstrated by the very low background luminescence with all PylRS variants in the absence of the UAA), as well as the high protein production capacity of the *Xenopus* embryo.^51^

The caged tyrosine **3** is poorly soluble in water, and attempts at injecting solutions with concentrations of 2.5 mM and above led to needle clogging. The tetrazine **6** on the other hand was soluble in water, but unexpectedly, injection did not lead to any appreciable incorporation into the Rluc reporter (this experiment has been repeated three times on different days). One possibility is that **6** might be able to diffuse out of the embryo to rapidly be diluted in the media. Thus, rather than injecting UAAs, we attempted soaking the embryos in media containing **1**–**6** (1 mM in embryo media, except for **3**, which was saturated at 0.25 mM) after injection of the RNAs. Embryos were incubated at RT in the presence or absence of the UAA until 24 hpf, and an Rluc assay was performed. Gratifyingly, the phenylalanine analogues **3** and **6** showed very good incorporation into the Rluc reporter after simple addition to the embryo water. In contrast, lysine analogues **1**, **2**, **4**, and **5** showed significantly better incorporation into the reporter with injection of the UAA (Supporting Figure S2).

For **3**, we measured a 120-fold increase in luminescence above the background, while **6** showed a 58-fold increase. We suspect that the phenylalanine backbone of UAA **3** and **6** may facilitate more efficient transport of the small molecule into cells to reach concentrations that achieved good expression of the Rluc reporter, in contrast to the lysine analogs. In *Xenopus* oocytes, phenylalanine has one of the highest rates of uptake from media by amino acid transporters as compared to other amino acids.^52^ For **6**, the particular synthetase mutant may not have been as efficient at aminoacylating tRNA as others, requiring a higher UAA concentration in order to observe adequate Rluc expression. Importantly, injection or incubation of the UAAs alone did not cause any toxicity beyond the background in embryos by 24 hpf (Supporting Figure S3). Overall, we successfully encoded six structurally and functionally diverse UAAs in the *Xenopus* embryo and achieved good protein expression levels that were comparable to those obtained via injection of wild-type Rluc mRNA (Supporting Figure S4). Expectedly, **1** had the highest rate of incorporation, followed by **5** > **2** > **3** > **4** = **6**. The efficiency of the synthetase mutants for acylating PylT with the UAA likely explains the differences in expression levels of the reporter. We have confirmed that the PylRS/PylT system is broadly applicable and highly orthogonal with six different PylRS variants, thus validating GCE as a universally effective strategy to add new chemistries to the genetic code of this animal model.

### Activating Protein Function with Light in *Xenopus* Embryos

**Figure 3:**
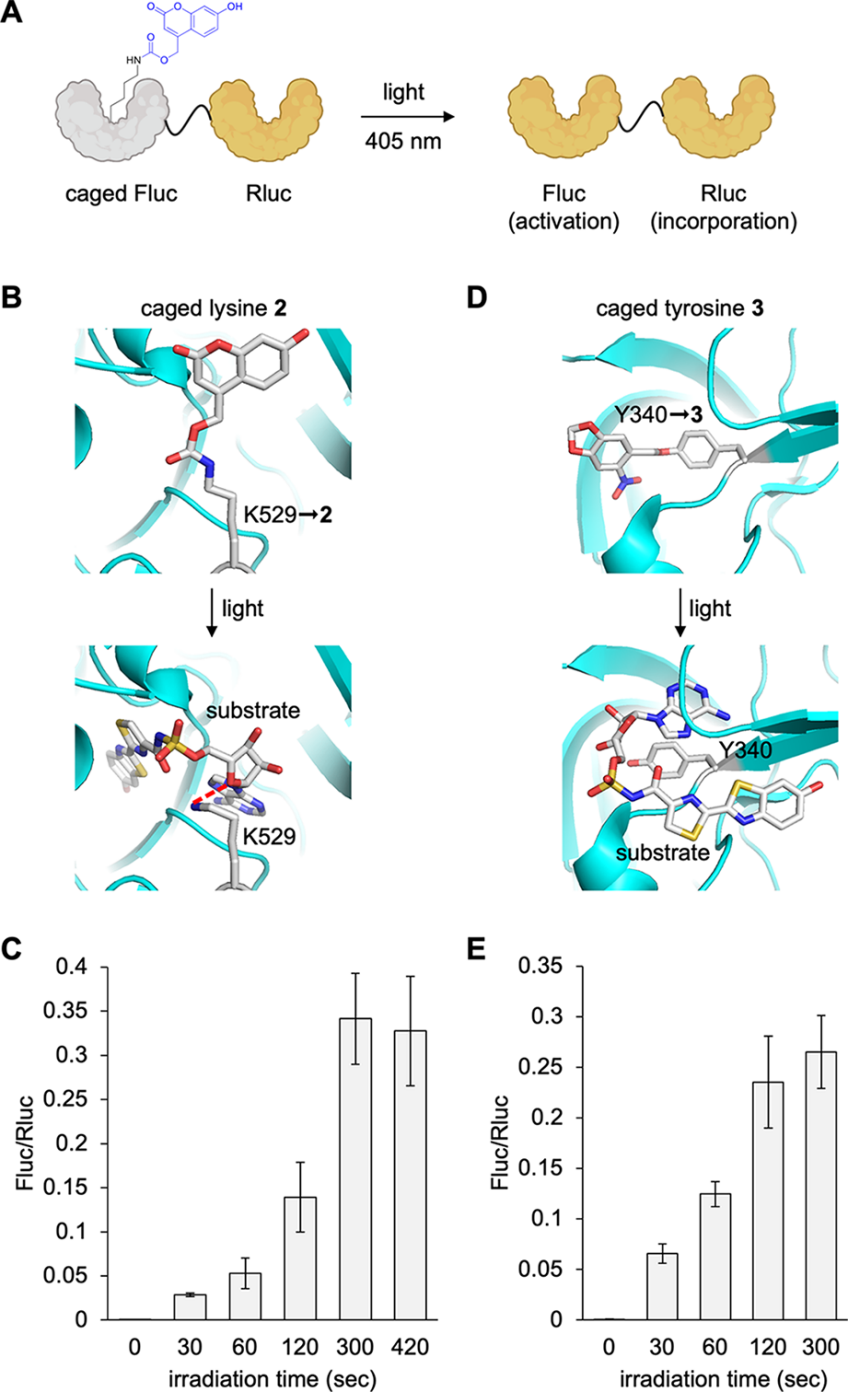
Conditional control of enzymatic activity with photocaged UAAs **2** and **3**. (A) Luciferase enzymatic activation in *Xenopus* embryos with light. Structural representation of caging of the Fluc active site with either (B) **2** or (D) **3** before and after irradiation (PDB: 4G36). Incorporation of (C) **2** or (E) **3** into the dual luciferase reporter and irradiation for increasing duration with a 405 nm light at 24 hpf. Bars represent means, and error bars represent standard deviations of measurements from three independent embryos.

Photocaged UAAs have been used to optically control proteins such as kinases,^6^ Cre recombinase,^53^ Cas9,^54^ caspase-3,^55^ and DNA helicase^40^ with temporal and spatial precision. Light-triggered activation of a protein is made possible by replacing an active site lysine or tyrosine residue with a photocaged analogue. The additional steric bulk of the caging group, accompanied by the altered electrostatic properties and loss of hydrogen bonding abilities of the caged amino or hydroxy group, blocks enzymatic function, until the cage is removed through irradiation with 405 nm light. To test the ability to optically activate enzyme function in *Xenopus* embryos, we used the aforementioned dual luciferase reporter with a C-terminal Rluc and an N-terminal firefly luciferase (Fluc). The Fluc gene contains a strategically placed amber stop codon at position K529, a critical lysine residue that hydrogen bonds with the adenylated luciferin intermediate and is important for catalysis (Figure 3A).^56^ Replacement of this lysine with **2** blocks luciferase function (Figure 3B), until photolytic cleavage of the caging groups restores the wild-type enzyme. Thus, the FlucK529TAG acts as a reporter for light activation, and Rluc acts as a reporter for amber stop codon suppression, enabling normalization of reporter function before and after optical stimulation and to account for a potential impact of light exposure on enzyme function. Embryos were injected with the mRNA and all other components, and at 24 hpf, they were irradiated with a 405 nm LED at increasing durations to determine the light dosage necessary for full activation of the reporter (Figure 3C).

Fluc activity, normalized to Rluc activity to account for differences in expression between embryos, plateaued by 5 min of irradiation, suggesting maximum activation. We observed a 584-fold increase in Fluc activity compared to nonirradiated samples. Virtually no background activity of the enzyme was observed in the absence of exposure to a 405 nm light. This is a distinct advantage of incorporation of caged amino acids for conditional control of enzyme function over optogenetics, where the optogenetic enzyme construct often has some activity in the “off” state.^57,58^ Another feature of optical control is the ability to readily tune protein activity through the titration of the light dose. We observed about 50% of max activity after a 2 min exposure, and about 15% activity after a 1 min irradiation. *Xenopus* embryos used here were pigmented, so light penetration could be hindered. In order to assess if pigmentation interferes in the decaging of **2**, we tested the irradiation and activation of Fluc after embryo lysis. The pigment, melanin, absorbs light in the UV and blue wavelength range,^59^ suggesting that it can impede light penetration significantly (Supporting Figure S5A), but is readily removed through centrifugation after embryo lysis. We saw maximal activation of Fluc activity within just 30 s, indicating that pigmentation may indeed be slowing down caging group photolysis (Supporting Figure S5B). The lysate irradiation led to a 670-fold increase in Fluc activity, comparable to *in vivo* irradiation. Thus, while pigmentation slows enzyme decaging, it does not prevent full decaging. In addition, these light doses did not cause any toxicity in embryos when irradiated at 6 hpf and incubated until 48 hpf (Supporting Figure S5C).

Photoactivation of caged tyrosine **3** was tested in a similar manner. Here, Y340 in Fluc was mutated to an amber stop codon. This site resides in the substrate binding pocket and is deemed to be essential for catalysis based on mutational analysis, as it is involved in binding and orienting the adenylated luciferin intermediate during catalysis (Figure 3D).^60,61^ An irradiation time course was conducted with live embryos, which showed Fluc activity plateauing by about 2 min of 405 nm exposure (Figure 3E). We again saw no activity of the caged enzyme before irradiation and excellent off-to-on switching (329-fold) through light exposure, suggesting caged tyrosine **3** as a useful UAA in the optical *Xenopus* toolbox.

### Activating Protein Function with Small Molecule Triggers in *Xenopus* Embryos

**Figure 4:**
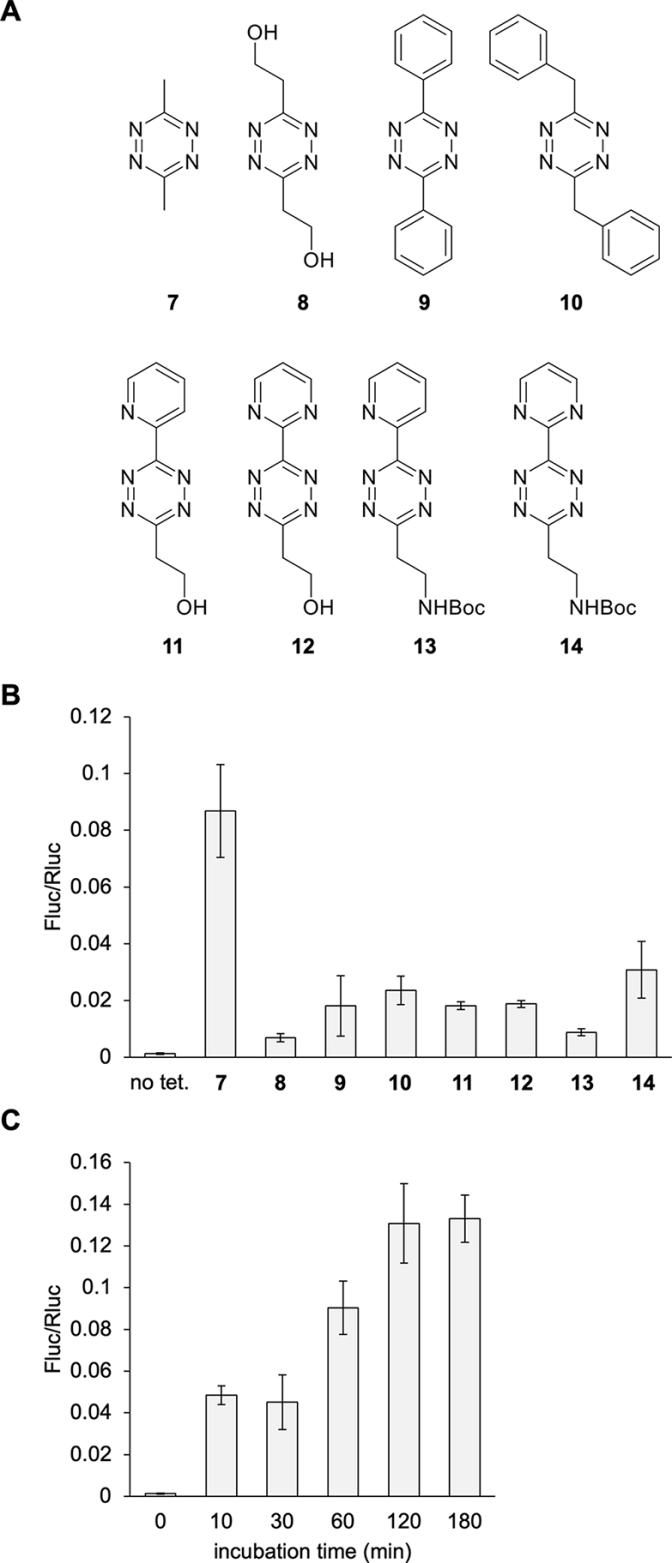
Conditional control of enzymatic activity through the incorporation of **4**. (A) Chemical structures of the different tetrazines tested for decaging of **4***in vivo*. (B) Tetrazine screen of dual-luciferase reporter activation. (C) Tetrazine **7** incubation timecourse. Bars represent mean, and error bars represent standard deviation of measurements from three independent embryos. No tet = no tetrazine control.

Caged UAAs that use small molecule triggers have been developed into general switches for conditional protein activation in cells and animals.^43,44,47,62^ The lysine derivative **4** is caged with a *trans*-cyclooctenyloxycarbonyl group. This group undergoes a bioorthogonal cycloaddition with a tetrazine, followed by spontaneous self-immolation to reveal lysine.^44^ The reaction between trans-cyclooctene and tetrazines is one of the fastest bioorthogonal ligation reactions;^63^ however, the self-immolation step is slower and dependent on the substituents on the tetrazine ring.^44^ Several tetrazine derivatives were tested for decaging **4** using the Fluc529TAG-Rluc reporter (Figure 4A). Live frog embryos at 24 hpf were incubated with each tetrazine at 100 μM for 1 h at RT. The tetrazine **7** showed the best activation of Fluc activity and appeared to plateau out by 2 h of incubation at RT with a 102-fold increase in Fluc activity compared to embryos not treated with tetrazine (Figure 4B,C). This was not a surprise, as this tetrazine was also observed to be efficient for decaging **4** in mammalian cells.^44^ Like the strategic placement of a photocaging group, incorporation of **4** completely abolishes Fluc enzymatic function.

Unfortunately, a toxicity assay of all tetrazines included in the screen showed that tetrazines **7** and **10** were both very toxic when embryos were treated at 24 hpf and then scored at 48 hpf (Supporting Figure S7). Tetrazine **9** and the asymmetric tetrazines were the next best candidates and showed no toxicity, albeit with a reduced decaging capacity of just a 14-fold increase. Tetrazine **9** may be a preferable option since it is commercially available. There is notable enzyme activation, but considerably less than the photocaged amino acids. Comparison of decaging of the same UAA with **7** or **9** has been performed before, with **7** removing 98% of the caging groups while **9** only removed 10% within a 3-h incubation period.^44^ Further exploration may reveal tetrazines with better decaging kinetics,^64^ while displaying no embryo toxicity.

**Figure 5:**
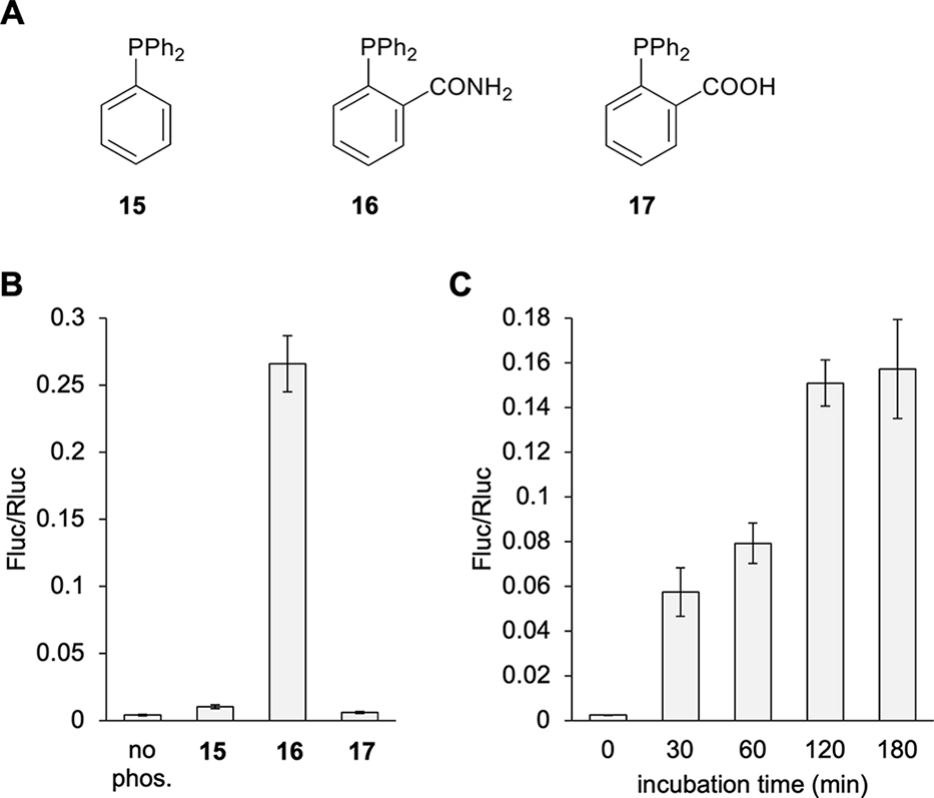
Conditional control of enzymatic activity through phosphine-based decaging of **5** incorporated into protein. (A) Chemical structures of the different phosphines tested. (B) Phosphine screen of dual-luciferase reporter activation. (C) Incubation timecourse with **16**. Bars represent mean, and error bars represent standard deviation of measurements from three independent embryos. No phos. = no phosphine control.

Another bioorthogonal reaction that has been used for decaging *in vitro* and *in vivo* is Staudinger reduction. Reduction of the azido group in **5** with a small molecule phosphine results in 1,6-elimination and decarboxylation, revealing lysine.^43^ We screened three phosphines for activation of the Fluc529TAG-Rluc reporter through simple addition to an embryo medium (Figure 5A). Aromatic phosphines were chosen for decaging of UAA **5** because aliphatic phosphines rapidly oxidize in aqueous buffers and reduce disulfide bonds in protein.^65,66^ Additionally, some aliphatic phosphines like TCEP are not cell permeable.^67−69^ Only phosphine **16** showed good activation of the reporter with a 65-fold increase in Fluc activity (Figure 5B). This was also the preferred phosphine in cell culture conditions.^43^ The reason for the reduced fold activation in comparison to the photocaged UAAs seemed to be a slightly increased basal activity of the caged Fluc before phosphine treatment. A phosphine treatment time course was conducted at 24 hpf with **16**, which showed enzyme activity to plateau by 2 h (Figure 5C). Since the phosphine can oxidize in aqueous conditions and showed a half-life of about 90 min in water,^70^ supplemented media were replaced at 90 min.

A toxicity assay for each phosphine was also conducted at 24 hpf using an extended 3 h incubation and phenotypic scoring at 48 hpf. All phosphines were observed to be nontoxic (Supporting Figure S7). Penetration of the developing embryo by the phosphine may be a limiting factor for fast activation of the reporter, so we also conducted the phosphine incubation timecourse in embryo lysate (Supporting Figure S8). Indeed, complete activation occurred within 30 min, supporting that phosphine penetration into the embryo was slower than decaging the protein itself. Additionally, the Fluc/Rluc ratio was higher than for the embryo incubation, suggesting that phosphine penetration was the cause of the lower than optimal Fluc activation. Unlike the tetrazine-activated *trans*-cyclooctene lysine **4**, near-complete decaging with small molecule treatment of the whole embryo was observed here, as can be seen when comparing assay results to non-TAG (wild-type) Fluc-Rluc reporter expression (Supporting Figure S8). Overall, when comparing the two small molecule activation approaches, the azide **5** stands out for its efficient incorporation (∼6-fold higher than **4**, Figure 2), the embryo’s tolerance to phosphine treatment, and improved off-to-on switching for the azide/phosphine pair **5**/**16** over the TCO/tetrazine pair **4**/**9**. While some background Fluc activity was observed in the absence of phosphine, it is very minor. A possible reason could be cytoplasmic reduction of the azide.^71^ In our case, the background reduction seems to be very slow, as 24 h incubation resulted in 99% of protein remaining caged. In summary, **5** makes an excellent addition to the genetic code expansion repertoire in *Xenopus* as a small molecule activated lysine with a mechanism that is orthogonal to light-induced decaging of **2** and **3**.

### Optical Control of GTPase Function in the *Xenopus* Embryo

**Figure 6:**
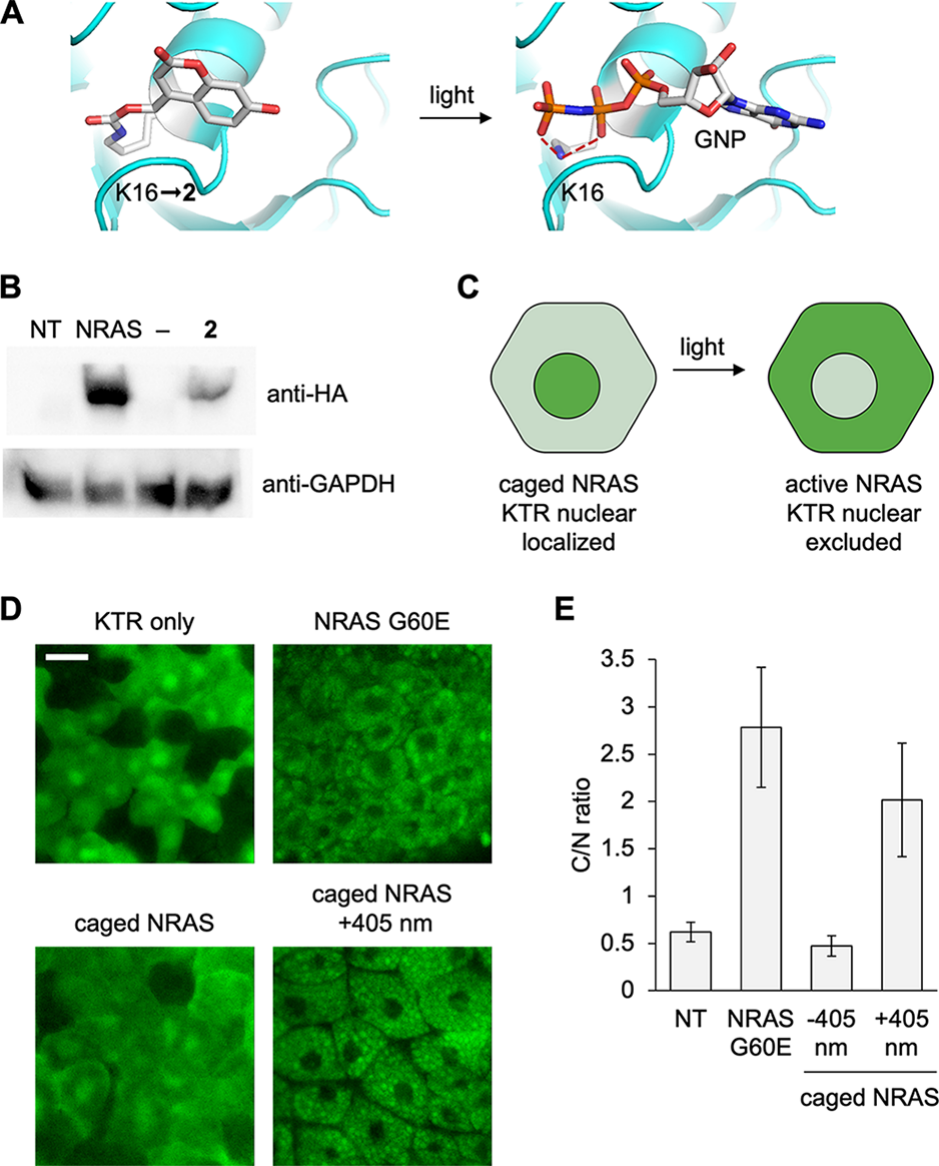
Optical control of NRAS activity using photocaged lysine **2**. (A) Structural representation of caging of the NRAS active site with **2** (PDB: 5UHV). (B) Western blot of incorporation of **2** into NRAS. (C) Caging of NRAS blocks its activity in the absence of light, and the ERKKTR-Clover reporter is nuclear localized. Irradiation at 405 nm activates NRAS, which leads to ERK activation and nuclear exclusion of the KTR. (D) Representative confocal images of the KTR reporter for each condition. Scale bar: 20 μm. (E) Quantification of the fluorescent cytosol/nucleus ratio. Bars represent means, and error bars represent standard deviations of measurements from 15 cells and three different embryos.

To demonstrate an application of the GCE methodology in frog embryos, we turned to NRAS, a GTPase in the RAS/MAPK signaling pathway (Supporting Figure S9). Many nucleotide binding pockets like those in kinases and GTPases contain a critical lysine residue that orients the nucleotide triphosphate by hydrogen bonding with the phosphate groups.^72^ Our lab and others have successfully caged the function of several kinases through this universal method.^6,25,73^ Here, we used a constitutively active NRAS mutant (G60E) that is associated with a family of diseases named the RASopathies,^74,75^ in order to decouple NRAS activation from upstream signaling. RASopathies make up a family of diseases characterized by congenital hyperactivating mutations of the RAS/MAPK pathway. These diseases disrupt a variety of developmental processes, from gastrulation to brain and heart development.^74,76−78^ We identified lysine 16 as the nucleotide binding lysine in the pocket (Figure 6A)^79^ and mutated it to an amber stop codon (K16TAG), then synthesized the corresponding mRNA through *in vitro* transcription.^80^ We incorporated the photocaged lysine **2** into the HA-tagged NRAS and performed Western blots to confirm the expression of the construct (Figure 6B). No background amber suppression was seen in the no UAA control, and a band at the same molecular weight as the non-TAG control was seen in the presence of **2**, confirming caged NRAS expression.

To assess activation of NRAS, we used an ERK kinase translocation reporter (KTR, Supporting Figure S10).^81^ This green fluorescent reporter is predominantly nuclear localized in the absence of RAS/MAPK signaling and translocates to the cytoplasm when active ERK is present (Figure 6C). Embryos expressing the caged NRAS and ERKKTR-Clover were incubated at 14 °C overnight and then irradiated at 20 hpf with 405 nm light and incubated for an additional hour. Animal cap explants were prepared for confocal imaging at stages 8–9 (Figure 6D).^82^ Nuclear localization of the reporter was seen in the KTR mRNA only injection and the caged NRAS expression in the absence of irradiation, confirming complete inactivity of the caged GTPase. Nuclear exclusion was seen in the constitutively active NRAS G60E control and after optical stimulation of embryos expressing the caged NRAS mutant, indicating RAS/MAPK activation. The cytoplasm/nucleus ratio (C/N) was calculated and averaged across three embryos per condition (Figure 6E). There was a significant 4-fold increase in the C/N ratio in the irradiated caged NRAS-expressing tissue compared to the embryos that were kept in the dark, further supporting fast activation of RAS/MAPK signaling. Overall, photocaging of NRAS activity was successful and showed excellent suppression of GTPase function and restoration of enzymatic activity upon light stimulation, thereby validating this approach for optical control of kinases and GTPases in *Xenopus* embryos. This is the first example of expression of a RASopathy mutant of NRAS in the *Xenopus* embryo.^83^

## Conclusions

In summary, we have demonstrated genetic code expansion in a new animal model, the *Xenopus* embryo, and achieved robust incorporation of six unique UAAs. Simple injection of RNAs encoding all required parts is a general approach for site-specific incorporation of the UAA into protein in live embryos. Incubating the embryo in UAA-supplemented media was an effective alternative method for UAA delivery when injection was not effective. This allowed us to engineer conditional control of enzymatic activity in *Xenopus* embryos using both light and small molecules as triggers to decage lysine or tyrosine side chains. These UAAs have wide ranging applicability because of the ubiquity of tyrosine and lysine residues in the active sites of enzymes and at protein–protein interface hotspots.^84,85^ Both residues routinely participate in hydrogen bonds to stabilize substrate–protein interactions. Lysine acts as a base in catalysis such as in proteases,^86^ can act as a nucleophile such as in histone methyltransferases,^87^ and can also form salt bridges. All UAAs completely blocked luciferase activity when placed in the active site and showed a reliable and predictable titration of enzymatic function when tuning irradiation or small molecule exposure time. While the embryo pigment did slow down the kinetics of photodecaging, quantitative activation of enzymatic activity was achieved. These caged UAAs work as irreversible switches for protein function, making them stand out from existing optogenetic constructs that require constant irradiation for activation. This is a desirable trait in the rapidly developing embryo, allowing for well-defined activation timing but minimal irradiation exposure. Both photocaged and small molecule-activated UAAs make excellent additions to the set of tools available for conditional control of protein function in the study of *Xenopus* biology. The *Xenopus* embryo model has many unique features that make it an attractive model for studying developmental biology. Useful high resolution imaging techniques, such as animal cap explants, are routinely used by *Xenopus* researchers. The well-defined fate mapping of early blastula stage embryos is also a unique feature of frog model system that could allow for targeted manipulation of specific tissue types in the animal. Taken together, expansion of the genetic code of *Xenopus* will be a useful tool for the study of embryo development and biology and how it relates to human disease. Future developments will involve studies with additional chemical functionalities, such as bioconjugation handles, fluorophores, biophysical probes, and photo-cross-linkers.

## Methods

### *Xenopus* Care and Microinjection

Embryos used in this study were obtained from a colony of *Xenopus laevis* frogs maintained at the University of Pittsburgh under the care of the Division of Laboratory Animal Research according to IACUC Protocol No. 18022377. *Xenopus laevis* embryos were obtained by in vitro fertilization using standard protocols.^88^ Fertilized eggs were dejellied in 50 mL of a 2% cysteine solution in 1/3 × modified Barth’s solution (MBS; pH 8) and were microinjected at the one-cell stage while in MBS supplemented with 4% Ficoll-400 (17–0300–05, GE Healthcare) using a microinjector (PLI-90, Harvard Apparatus).^89^ Embryos were transferred to 1/3 × MBS supplemented with antibiotic and antimycotic (100 units/ml penicillin, 0.1 mg mL^–1^ streptomycin, and 0.25 μg/mL amphotericin B, Sigma) and incubated in 35 mm Petri dishes at RT, unless otherwise specified. All *Xenopus laevis* frogs used in this study were wild type pigmented and obtained commercially (Nasco, Fort Atkinson, WI). For genetic code expansion injections, injection solutions were prepared on ice in 1.5 mL microcentrifuge tubes. A solution of 50 ng/μL PylRS mRNA, 50 ng/μL Fluc-Rluc or NRAS mRNA, 3 μg/μL PylT, and 10 mM UAA (from 100 mM stock) was prepared in a total of 4 μL. Thus, a total of 250 pg of the PylRS mRNA, 250 pg of the Fluc UAG-Rluc reporter or NRAS 16TAG mRNA, 15 ng of PylT, and 50 pmol of UAA were injected in a volume of 5 nL into the center of the cell at the one-cell stage. Phenol red was added at a final concentration of 0.05% in injection solutions as a tracer. For cases where the UAA was added to the media, a final concentration of 1 mM UAA (0.25 mM for **3**) was added to 1/3 MBS (500 μL), and 10–20 embryos were incubated in a 24-well plate until the desired time point. For NRAS G60E, ERK-KTR, or WT Fluc-Rluc, 50 pg was injected.

### Embryo Irradiation and Compound Treatment

For embryo irradiation, a 405 nm LED (Luxeonstar, Luxeon Z, 675 mW) was placed 3 cm above the 35 mm Petri dish containing the embryos suspended in 1/3 × MBS. Light output at the specimen was measured at 350 mW with a Thorlabs power meter. For tetrazine treatment experiments, a 100 mM stock in DMSO was made for each tetrazine which was then diluted to 100 μM in 1/3 × MBS (1 μL in 1 mL of 1/3 × MBS). For phosphine treatment studies, 50 mM stock solutions of each phosphine in DMSO were diluted to 50 μM for phosphine **15** (1 μL in 1 mL) and 100 μM for **16** and **17** in 1/3 × MBS (2 μL in 1 mL). Embryos (*N* = 10–20) were suspended in 500 μL of the compound solution in a 24 well plate and incubated on the benchtop at RT (23 °C) protected from light by aluminum foil for the desired time. For phosphine incubations, the phosphine supplemented medium was replaced with fresh phosphine supplimented medium at 90 min. At the end of the incubation time, the medium was removed, and embryos were suspended in fresh 1/3 × MBS. For luciferase assays, for each condition, three embryos were collected, each in individual 1.5 mL microcentrifuge tubes at 24 hpf. The remaining water was removed, and 50 μL of passive lysis buffer (Promega) was added to each tube. Embryos were manually homogenized with a p200 pipet tip. Samples were centrifuged at 16 200 rcf for 8 min at 4 °C. A volume of 30 μL of lysate was added to a white-bottom 96-well plate and loaded into a plate reader with autoinjection function (Tecan Infinite M1000 Pro, 200 μL/sec). A *Renilla* Luciferase Assay kit or Dual Luciferase Assay kit (Promega) was used for the assay. For the Rluc 95TAG assays, 20 μL of Rluc substrate was injected per well and a luminescence reading was taken 2 s later. For the dual luciferase assays, an assay program was used to inject 20 μL of Fluc assay reagent, paused 2 s, a reading taken, then 20 μL of the Stop and Glo Rluc assay reagent injected, paused 2 s, and a reading taken. Autoattenuation mode was on. Corrected Fluc values were calculated by dividing each individual Fluc value by its Rluc value (Fluc/Rluc). Standard deviation was calculated from three independent samples per condition to represent the error bars in graphs.

### Animal Cap Explants and Imaging

Between three and five embryos per condition were selected at early gastrula (stage 10) and transferred into 10 mL of Danilchik’s For Amy (DFA) medium supplemented with antibiotics and antimycotics (100 units/ml penicillin, 0.1 mg mL^–1^ streptomycin, and 0.25 μg/mL amphotericin B, Sigma) in a 5 cm Petri dish. The vitelline membranes were manually removed with sharpened forceps under a dissecting stereomicroscope (10×), and animal cap explants were microsurgically excised using hair tools. The explants were placed in fresh DFA in 35 mm glass bottom dishes, held in place by a coverslip placed on top of the explants. A Zeiss LSM 700 laser scanning confocal microscope was used for imaging with a 488 nm laser (20× water immersion lens; 1024 × 1024 pixel frame; 40–80% laser power; gain, 900; pinhole, 1.5 airy units; one scan; 15 μs pixel dwell time). Fluorescent cytoplasm/nucleus (C/N) intensity ratios were calculated by using ImageJ software. Background-subtracted measurements of fluorescent intensity were taken from selected regions in the nucleus and cytoplasm for 15 cells across three independent explants per condition. This was performed by measuring the mean gray value of the nuclear or cytoplasmic regions, subtracting the mean gray value of the background (from an area outside the embryo), and then calculating the ratio for each cell independently.

### Western Blot from *Xenopus* Embryo Lysate

Embryos (10) were incubated at 14 °C until 24 hpf and then collected in a 1.5 mL microcentrifuge tube for each condition. Next, 200 μL of HEPES buffer (20 mM, pH 7.3), supplemented with protease inhibitor (Roche cOmplete, Mini Protease Inhibitor Cocktail), was added, and embryos were lysed by pipetting up and down with a p200 tip about 15 times.^90^ The yolk platelets were pelleted by centrifugation at 800 rcf for 10 min, and the supernatant was collected into a new microcentrifuge tube. Lysates were centrifuged at 16 200 rcf for 5 min at 4 °C, and 30 μL of lysate was mixed with 10 μL of 4× SDS loading buffer, denatured at 95 °C for 5 min, and loaded onto a 10% SDS polyacrylamide gel for SDS-PAGE (150 V for 90 min). Protein was transferred to a PDVF membrane (80 V for 90 min), blocked with 5% BSA in TBST for 1 h at RT, and incubated with either anti-HA rabbit monoclonal antibody (1:1000, CST #3724) or anti-GAPDH rabbit polyclonal antibody (1:1000, Proteintech 50−172−604 6351) in 5% BSA TBST overnight at 4 °C. Blots were washed three times with TBST and incubated with antirabbit monoclonal antibody-HRP (1:1000 CST #7074) in TBST at RT for 1 h. Blots were washed three times with TBST and then developed with SuperSignal West Pico Chemiluminescent Substrate (Thermo) for 5 min before chemiluminescent imaging on a ChemiDoc imaging system (Bio-Rad).

## Supporting Information Available

Experimental methods and supporting figures, including luciferase assays and toxicity assays (PDF)
