# Supplementary material for: Expanding the Genetic Code of Xenopus laevis Embryos
Source: ACS Chem Biol. 2024 Jan 26;19(2):516–25. doi: 10.1021/acschembio.3c00686 (PMC10877573; doi:10.1021/acschembio.3c00686)
Supplement: Supplementary file 1 — cb3c00686_si_001.pdf [file cb3c00686_si_001.pdf]

Supporting Information

Expanding the Genetic Code of *Xenopus Laevis* Embryos

Wes Brown[a], Lance Davidson[b], and Alexander Deiters*[a]

[a] Department of Chemistry, University of Pittsburgh, Pittsburgh, PA 15260, United States

[b] Departments of Bioengineering, Developmental Biology, and Computational and Systems Biology, University of Pittsburgh, Pittsburgh, PA, 15260, United States

Contact information: deiters@pitt.edu, lad43@pitt.edu

Experimental Methods

Plasmid construction

For cloning of the TetF PylRS (**Table S1**) into pCS2, amplification of the insert was performed using primers 1 and 2 (**Table S2**) in a Taq polymerase PCR (50 µl reaction, Thermo): 34 µl of MilliQ water, 5 µl of 10x Taq buffer, 3 µl of $MgCl_2$ (25 mM), 2.5 µl of each primer (100 µM), 1 µl of dNTP mix (10 mM), 1 µl of DNA template (10 ng/µl), and lastly 1 µl of Taq polymerase (5 U/µl).The following cycling program was used: 95 °C for 3 min, 34 cycles of 95 °C for 30 sec, 55 °C for 30 sec, 72 °C for 60 sec, then 72 °C for 5 min. The PCR reaction was purified with the PCR cleanup kit (Thermo). The pCS2 backbone (4 µg) and amplified insert were digested with BamH1 and Xho1 (NEB, 0.5 µl of each, 1 µl of 10x Cutsmart buffer, MilliQ water up to 10 µl) for 1 hour at 37 °C. The entire reaction was loaded onto a 0.8% agarose-TBE gel (40 ml) with 0.2 µg/ml ethidium bromide and electrophoresed for 40 minutes at 80 volts in TBE buffer. The linearized vector (4000 bp) and insert (1389 bp) were excised followed by gel extraction using the GeneJET Gel Extraction kit (Thermo) according to the manufacturer's protocol. The TetF PylRS gene (37.5 ng, 2 molar eq. of backbone) was incubated with digested pCS2 (50 ng) in a 10 µL reaction with 1 µl T4 DNA Ligase (400 U/µl) and 1x T4 DNA ligase buffer (NEB) at room temperature for 1 hour. Top10 chemically competent cells were transformed with 5 µl of the T4 DNA ligase reaction in the same manner as detailed above. Plating, colony selection, miniprep, and sequence validation followed the same steps as detailed above to yield pCS2-TetF PylRS.

The FlucY340TAG-Rluc reporter was cloned into pCS2 using Gibson assembly. The pCS2 backbone (4 µg) was digested with Bamh1 and EcoR1 (NEB, 0.5 µl of each, 1x Cutsmart buffer) in a 10 µL reaction for 1 hour at 37 °C. The product was loaded onto a 0.8% agarose-TBE gel and electrophoresed for 40 minutes at 80 volts in TBE buffer, and the linearized vector (4000 bp) was excised followed by gel extraction using the GeneJET Gel Extraction kit (Thermo) according to the manufacturer's protocol. The Fluc Y340TAG gene were amplified with primers 3 and 4, and Rluc was amplified with primers 5 and 6 using Taq polymerase PCR (50 µl reaction, Thermo) following the manufacturer protocol. The following cycle conditions were used: 95 °C for 3 min, 34 cycles of 95 °C for 30 sec, 55 °C for 30 sec, 72 °C for 60 sec, then 72 °C for 5 min. The gene product was purified by agarose gel electrophoresis and gel extraction as mentioned above. Next, the purified FlucY340TAG gene and Rluc gene fragments were used in Gibson assembly reactions with 100 µg of pCS2 digested vector and 1 molar equivalent of each insert fragment mixed in a total volume of 5 µl, then added to 15 µl of Gibson assembly master mix (20 µL, final concentrations: 1x isothermal reaction buffer (100 mM Tris-HCl pH 7.5, 10 mM $MgCl_2$, 0.2 mM of each dNTP, 10 mM DTT, 5% PEG–80 00, 1 mM NAD^+), 4U/µl Taq DNA ligase, 4 U/ml T5 exonuclease, and 25 U/ml Phusion DNA polymerase) then incubated at 50 °C for 1 hour. Top10 chemically competent cells (50 µL) were heat shock transformed (45 seconds at 42 °C, then 2 minutes on ice) with 5 µL of the Gibson assembly reaction, rescued with 200 µl of SOC media, all 250 µl plated on ampicillin (100 µg/ml) containing LB agar, and colonies were selected after overnight incubation at 37 °C. Overnight cultures in 5 ml of LB media containing ampicillin were miniprepped using the GeneJET Plasmid Miniprep kit (Thermo) following the manufacturer's protocol to yield pCS2-FlucY340TAG-Rluc. Sequence confirmation of the construct was performed by Sanger sequencing using SP6 and T3 primers (Genewiz, standard primers).

In vitro transcription

In order to produce mRNA, 4 µg of the pCS2 plasmid was linearized with Not1 (NEB, 1 µl) in a 10 µL reaction with 1x cutsmart buffer at 37 °C for 1 hour in a 1.5 ml microcentrifuge tube. The reaction mixture was diluted with water to a total volume of 50 µL and purified by phenol:chloroform:isoamyl alcohol (PCIA, 50 µL) extraction. The tube was vortexed, then centrifuged at 16,200 rcf for 1 minute. The top aqueous layer was carefully pipetted into a fresh 1.5 ml microcentrifuge tube. Next, 5 µl of a 3 M sodium acetate solution in water (1/10 total volume) was added and the mixture was vortexed. The linearized DNA was then precipitated by adding ethanol (138 µL, 2.5x total volume) and incubating at –20 °C overnight, followed by pelleting at 16,200 rcf for 5 minutes. The DNA pellet was washed with 70% ethanol (200 µl), centrifuged again at 16,200 rcf for 2 minutes, the supernatant was removed carefully without disruption of pellet, and then the pellet was dried at room temp with the cap open for no more than 5 minutes before dilution in 10 µL of MilliQ water. The linearized plasmid (1 µg) was used in a Sp6 mMessage mMachine *in vitro* transcription reaction at 37 °C for 2 hours following manufacturer instructions (Invitrogen). The reaction mixture contained linearized DNA template (1 µg), NTP/cap mix (10 µL), 10x buffer (2 µL), and SP6 RNA polymerase (2 µL) and was diluted up to a final volume of 20 µL with MilliQ water. Reaction mixtures were incubated at 37 °C for 1-2 hours. Reaction mixtures were treated with 1 µL of Turbo DNase (Thermo) for 15 min at 37 °C, purified by PCIA extraction, ethanol precipitated, and dissolved in 20 µl of MilliQ water as described above and stored at –80 °C. RNA concentration was measured by diluting the 1 µl of RNA into 9 µl of RNase free water, mixing, and measuring absorbance at 260 nm using a spectrophotometer (Nanodrop). That diluted sample was then loaded onto a 0.8% agarose gel in TBE buffer and electrophoresed (80 V, 40 min) along with TriDye DNA ladder (NEB) to check on RNA quality.

To make PylT, first the PylT template was amplified with a U25C mutation and CCA at the 3' end to increase aminoacylation efficiency (Table 1),[1] through PCR: 34 µl of MilliQ water, 5 µl of 10x Taq buffer (100 mM Tris-HCl (pH 8.8), 500 mM KCl), 3 µl of $MgCl_2$ (25 mM), 2.5 µl of each primer (100 µM), 1 µl of dNTP mix (10 mM), 1 µl of DNA template (10 ng/µl), and lastly 1 µl of Taq polymerase (5 U/µl). PCR primers were used to install a truncated T7 promotor at the 5' end. The following thermocycler settings were used: 95 °C for 3 minutes followed by 34 cycles of 95 °C for 30 seconds, 45 °C for 30 seconds, then 72 °C for 90 seconds, then finished with 72 °C for 5 minutes. The PCR product was purified by PCIA extraction and ethanol precipitation as mentioned above. A total of 1 µg of this purified PCR product was used in a T7 MegaScript in vitro transcription reaction (Thermo) incubated at 37˚C for 5 hours. The reaction mixture contained DNA template (1 µg), NTPs (10 µL), 10x buffer (2 µL), and T7 RNA polymerase (2 µL) and was diluted up to a final volume of 20 µL with MilliQ water. Reaction mixtures were incubated at 37 °C for 5 hours. At the end of the reaction, TurboDNase (1 µl) was added and incubated for 15 minutes at 37 °C. The product was purified with PCIA extraction, then ethanol precipitation as detailed above. The PylT was refolded using a thermocycler: 95 °C for 1 minute, then 5 °C drops every 15 seconds until the temperature reaches 10 °C. A 1/10 dilution with MilliQ water was made and the RNA concentration was checked with a Nanodrop. The quality of the PylT was assessed by agarose gel electrophoresis. The rest of the diluted RNA solution was loaded onto a 40 ml 1.5% agarose-TBE gel containing 0.2 µg/ml ethidium bromide alongside a DNA ladder (FastRuler Ultra Low Range DNA Ladder, Thermo) and was electrophoresed for 40 minutes at 80 V in TBE buffer, then imaged under UV light. Expect a single band around 100 bp if the transcription reaction was successful. No band or smearing would suggest failure of transcription or degradation of product.

Synthesis and preparation of chemical reagents

All UAAs were synthesized according to published protocols, except for **1** which is commercially available (Chem Impex).[2-6] UAAs **1**, **2**, and **6** were prepared at 100 mM solutions in water and UAAs 3-5 were prepared in 100 mM solutions in DMSO. All were stored as 50 µl aliquots at –20 °C. The phosphine 15 was purchased from Chem-Impex (50-495-422). The phosphines **16**[7] and **17**[8] were synthesized according to published protocols. The tetrazines **7**,[9] **8**,[10] **9**,[11] **10**,[12] and 11-14[13] were synthesized according to published protocols. The phosphines were all prepared as 50 mM solutions in DMSO and stored at –20 °C. All tetrazines were prepared as 100 mM aliquots in DMSO and stored at –20 °C.

Supporting Tables

Name	UAA	270	271	274	311	313	315	349
PylRS	AllocK (**1**)	L	Y	L	N	C	M	Y
HCKRS	HCK (**2**)	L	**A**	**M**	N	C	M	Y
OABKRS	TCOK (**3**)	L	**A**	L	N	C	M	**F**
PABKRS	PABK (**4**)	L	Y	**A**	N	**A**	M	**F**
NPYRS	NPY (**5**)	**F**	Y	**M**	**G**	**G**	M	**F**
TetFRS	TetF (**6**)	**G**	Y	L	**G**	**S**	M	Y

Table S1. Synthetases used in this study with binding pocket mutations bolded.

Primer	Sequence (5' to 3')
1	CATTAGGATCCATGGATAAAAAACCGCTGGATGTGCTGATTAGC
2	CTAATACTCGAGTTACAGGTTCGTGCTAATGCCGTTATAGTAGC
3	ATAGAATACAAGCTACTTGTTCTTTTTGCAGGATCCATGGAAGACGCCAAAAACATAAAGAAAGGCCC
4	ACCACCGCCAGAACCGCCGCTACCGCCGCCCACGGCGATCTTTCCGCCCTTC
5	GGCGGCGGTAGCGGCGGTTCTGGCGGTGGTATGGCTTCCAAGGTGTACGACCCC
6	CACTATAGTTCTAGAGGCTCGAGAGGCCTTGAATTCTTACTGCTCGTTCTTCAGCACGCG

Table S2. Names and sequences of primers used in this study.

Supporting Figures

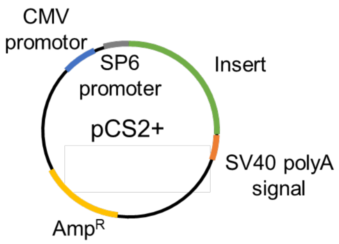

Inserts

Synthetases
- PylRS (WT)
- HCKRS
- TCOKRS
- PABKRS
- NPYRS
- TetFRS

Luciferase reporters
- Rluc WT
- Rluc L95TAG
- Fluc-Rluc (WT)
- Fluc K529TAG-Rluc
- Fluc Y340TAG-Rluc
- NRAS G60E
- NRAS G60E K16TAG
- ERKKTR clover

Figure S1. Plasmid map of constructs used in this study.

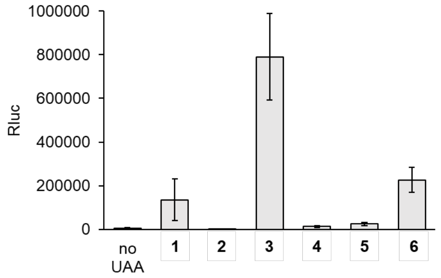

Figure S2. Luciferase assays after addition of UAAs to embryo media. UAAs were added to *Xenopus* embryo media at a 1 mM final concentration (0.25 mM for 3) after injection of the Rluc95UAG mRNA, PylRS mRNA, and PylT. Embryos were incubated at 23 °C and assays were conducted at 24 hpf.

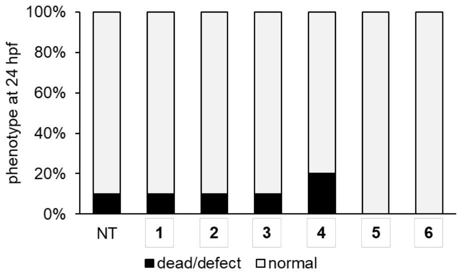

Figure S3. Toxicity assay of UAAs **1-6** in xenopus embryos measured at 24 hpf. A volume of 5 nl of a 10 mM solution of UAAs **1**, **2**, **4**, and **5** were injected and embryos were incubated at 23 °C for 24 hours. For UAA **3**, embryos were incubated at 23 °C in embryo media supplemented with 0.25 mM of **3**. For UAA **6**, embryos were incubated at 23 °C in embryo media supplemented with 1 mM of **6**. N = 10 for each condition.

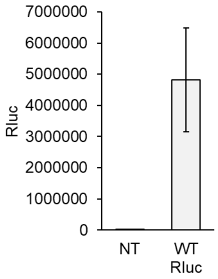

Figure S4. Assay after injection of WT Rluc mRNA (50 pg). Injection of 50 pg wild-type Rluc mRNA, incubation at 23 °C for 24 hpf, then assay of three embryos per condition. Bars represent mean and error bars represent standard deviation. NT = non-injected embryo control.

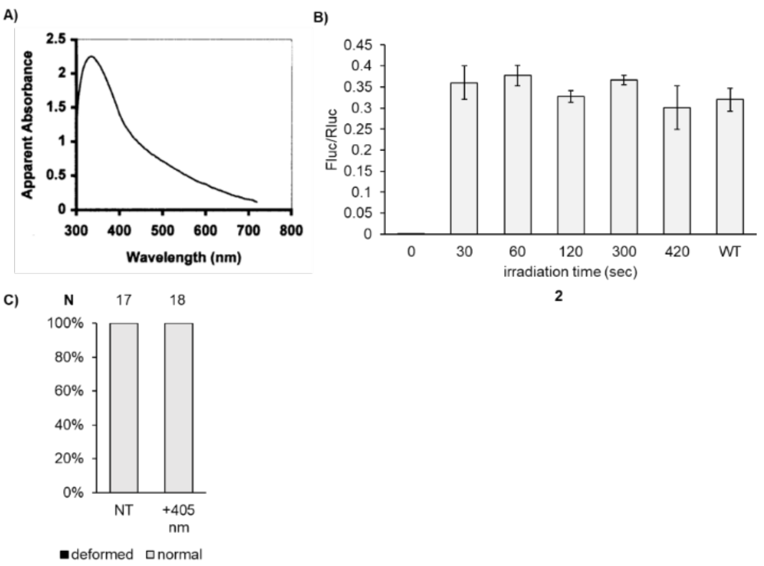

Figure S5. Activation of UAA **2** incorporated dual-luc reporter using 405 nm light after embryo lysis. A) Absorption spectrum of melanin. Adapted from Antony, R. Y., Chromophores in human skin. Physics in Medicine & Biology 1997, 42 (5), 789. B) Fluc activation from irradiation of lysate conducted after embryo lysis at 24 hpf. Bars represent means and error bars represent standard deviations of biological triplicates. C) Toxicity assay of 405 nm light irradiation at 6 hpf for 5 min, then scoring of embryos at 48 hpf. N = number of embryos for each condition.

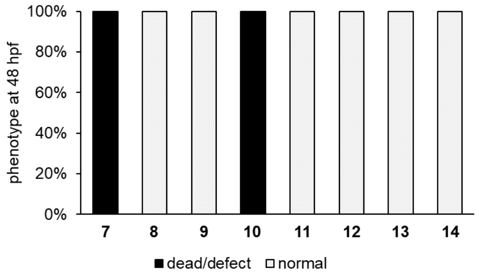

Figure S6. Toxicity assay of the tetrazines after exposure for 3 hours at 24 hpf and scoring at 48 hpf. Concentration for each tetrazine was 100 μM in embryo media. N = 15 for each condition.

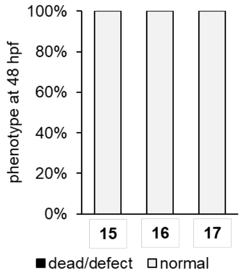

Figure S7. Toxicity assay of phosphines after treatment at 48 hpf. Concentration was 50 μM for **15** and 100 μM for **16** and **17**. Embryos were treated at 24 hpf for 3 hours and then scored at 48 hpf. N = 20 for each condition.

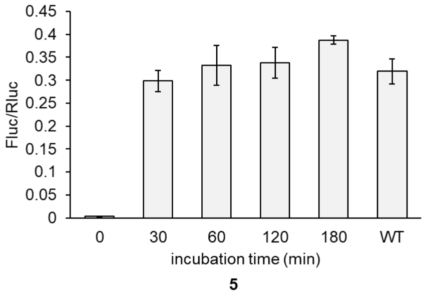

Figure S8. Activation of UAA **5** incorporated dual-luc reporter using phosphine after embryo lysis. Phosphine **16** (100 μM) was added to the lysate and incubated at 23 °C for the time indicated. Bars represent mean and error bars represent standard deviation of biological triplicate.

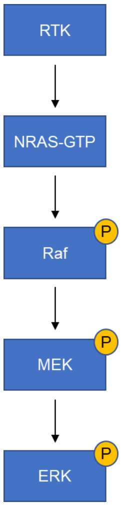

Figure S9. Schematic of the signaling pathways involving NRAS. Phosphorylation of ERK leads to gene transcription.

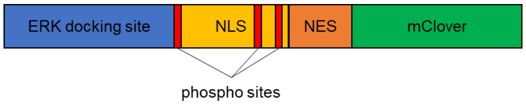

Figure S10. Schematic of the ERK-KTR reporter. In its unphosphorylated state, the nuclear localization predominates. Phosphorylation of the NLS sites by ERK inactivates the NLS sequence, allowing for nuclear exclusion to predominate.

References

1. Liu, J.; Hemphill, J.; Samanta, S.; Tsang, M.; Deiters, A., Genetic Code Expansion in Zebrafish Embryos and Its Application to Optical Control of Cell Signaling. *Journal of the American Chemical Society* **2017,** *139* (27), 9100-9103.
2. Luo, J.; Uprety, R.; Naro, Y.; Chou, C.; Nguyen, D. P.; Chin, J. W.; Deiters, A., Genetically encoded optochemical probes for simultaneous fluorescence reporting and light activation of protein function with two-photon excitation. *Journal of the American Chemical Society* **2014,** *136* (44), 15551-15558.
3. Luo, J.; Torres-Kolbus, J.; Liu, J.; Deiters, A., Genetic Encoding of Photocaged Tyrosines with Improved Light-Activation Properties for the Optical Control of Protease Function. *ChemBioChem* **2017,** *18* (14), 1442-1447.
4. Li, J.; Jia, S.; Chen, P. R., Diels-Alder reaction–triggered bioorthogonal protein decaging in living cells. *Nature chemical biology* **2014,** *10* (12), 1003-1005.
5. Wesalo, J. S.; Luo, J.; Morihiro, K.; Liu, J.; Deiters, A., Phosphine-Activated Lysine Analogues for Fast Chemical Control of Protein Subcellular Localization and Protein SUMOylation. *Chembiochem* **2020,** *21* (1-2), 141-148.
6. Jang, H. S.; Jana, S.; Blizzard, R. J.; Meeuwsen, J. C.; Mehl, R. A., Access to Faster Eukaryotic Cell Labeling with Encoded Tetrazine Amino Acids. *Journal of the American Chemical Society* **2020,** *142* (16), 7245-7249.
7. Saneyoshi, H.; Ochikubo, T.; Mashimo, T.; Hatano, K.; Ito, Y.; Abe, H., Triphenylphosphinecarboxamide: An Effective Reagent for the Reduction of Azides and Its Application to Nucleic Acid Detection. *Organic Letters* **2014,** *16* (1), 30-33.
8. Kim, Y. K.; Lee, S. J.; Ahn, K. H., New Hybrid Ligands with a trans-1,2-Diaminocyclohexane Backbone: Competing Chelation Modes in Palladium-Catalyzed Enantioselective Allylic Alkylation. *The Journal of Organic Chemistry* **2000,** *65* (23), 7807-7813.
9. Versteegen, R. M.; Rossin, R.; ten Hoeve, W.; Janssen, H. M.; Robillard, M. S., Click to Release: Instantaneous Doxorubicin Elimination upon Tetrazine Ligation. *Angewandte Chemie International Edition* **2013,** *52* (52), 14112-14116.
10. Yang, J.; Karver, M. R.; Li, W.; Sahu, S.; Devaraj, N. K., Metal-Catalyzed One-Pot Synthesis of Tetrazines Directly from Aliphatic Nitriles and Hydrazine. *Angewandte Chemie International Edition* **2012,** *51* (21), 5222-5225.
11. Hu, W.-X.; Rao, G.-W.; Sun, Y.-Q., Synthesis and antitumor activity of s-tetrazine derivatives. *Bioorganic & Medicinal Chemistry Letters* **2004,** *14* (5), 1177-1181.
12. Feng, H.; Zhang, H.; Wang, M.; Vannam, R.; Wang, H.; Yan, X.; Ouyang, W.; Jia, X.; Fox, J. M.; Li, Z., Improving Tumor-to-Background Contrast through Hydrophilic Tetrazines: The Construction of 18F-Labeled PET Agents Targeting Nonsmall Cell Lung Carcinoma. *Chemistry – A European Journal* **2020,** *26* (21), 4690-4694.
13. Sarris, A. J. C.; Hansen, T.; de Geus, M. A. R.; Maurits, E.; Doelman, W.; Overkleeft, H. S.; Codée, J. D. C.; Filippov, D. V.; van Kasteren, S. I., Fast and pH-Independent Elimination of trans-Cyclooctene by Using Aminoethyl-Functionalized Tetrazines. *Chemistry – A European Journal* **2018,** *24* (68), 18075-18081.
